# The Cephalic Morphology of *Protopselaphus*—A Phylogenetic Connecting Link in Staphylinidae (Coleoptera)

**DOI:** 10.1002/jmor.70167

**Published:** 2026-09-01

**Authors:** Margarita I. Yavorskaya, Paweł Jałoszyński, Shûhei Nomura, Rolf G. Beutel

**Affiliations:** ^1^ Institute of Evolution and Ecology University of Tübingen Tübingen Germany; ^2^ Museum of Natural History University of Wrocław Wrocław Poland; ^3^ National Museum of Nature and Science Tokyo Japan; ^4^ Institut für Zoologie und Evolutionsforschung, Friedrich‐Schiller‐Universität Jena Jena Germany; ^5^ Entomology II, Senckenberg Forschungsinstitut und Naturmuseum Frankfurt Frankfurt Germany

**Keywords:** 3D‐reconstruction, anatomy, head, micro‐CT, *Protopselaphus*, rove beetles

## Abstract

The head morphology of an ethanol‐preserved specimen of *Protopselaphus* was examined using synchrotron µCT and SEM. The morphology, including muscles, nervous system, digestive tract and glands, is described in detail for the first time. We interpret the observed external and internal features with respect to their possible phylogenetic implications, mainly focused on the groundplan of the clade Protopselaphinae + Pselaphinae, and on apomorphies of the latter group. Whereas a clade Dasycerinae + (Protopselaphinae + Pselaphinae) is still not strongly supported morphologically, the monophyly of Protopselaphinae + Pselaphinae is robustly established. Adult cephalic synapomorphies are vertical tentorial columns completely detached from the tentorial bridge and dorsally firmly connected with the head capsule, a brain shifted to the narrowed neck region and filling out this space almost completely, and strongly developed labio‐hypopharyngeal glands. Predacious habits and correlated falcate mandibles are also likely a groundplan apomorphy of this clade. Enlarged maxillary palps are arguably a typical feature but this condition varies strongly in the subfamily and also within Staphylinidae. Pselaphinae are characterized by several cephalic apomorphies such as a thin (or anteriorly obliterated, or missing) anterior tentorial arm, an antennal insertion covered by a frontal projection, a labral gland, a thin apical sensillum on the terminal maxillary palpomere, a highly reduced labial palpomere 3, and M. frontopharyngalis posterior composed of only one subcomponent.

## Graphical abstract

The head morphology including soft tissues of an ethanol‐preserved specimen of *Protopselaphus* was examined using synchrotron‐µCT and SEM and interpreted with respect to their possible phylogenetic implications. Our findings strongly support the monophyly of Protopselaphinae + Pselaphinae, with adult cephalic synapomorphies including the vertical tentorial columns completely detached from the tentorial bridge, a brain shifted to the narrowed neck region, and strongly developed labio‐hypopharyngeal glands. Predacious habits and correlated falcate mandibles are also likely a groundplan apomorphy of this clade.

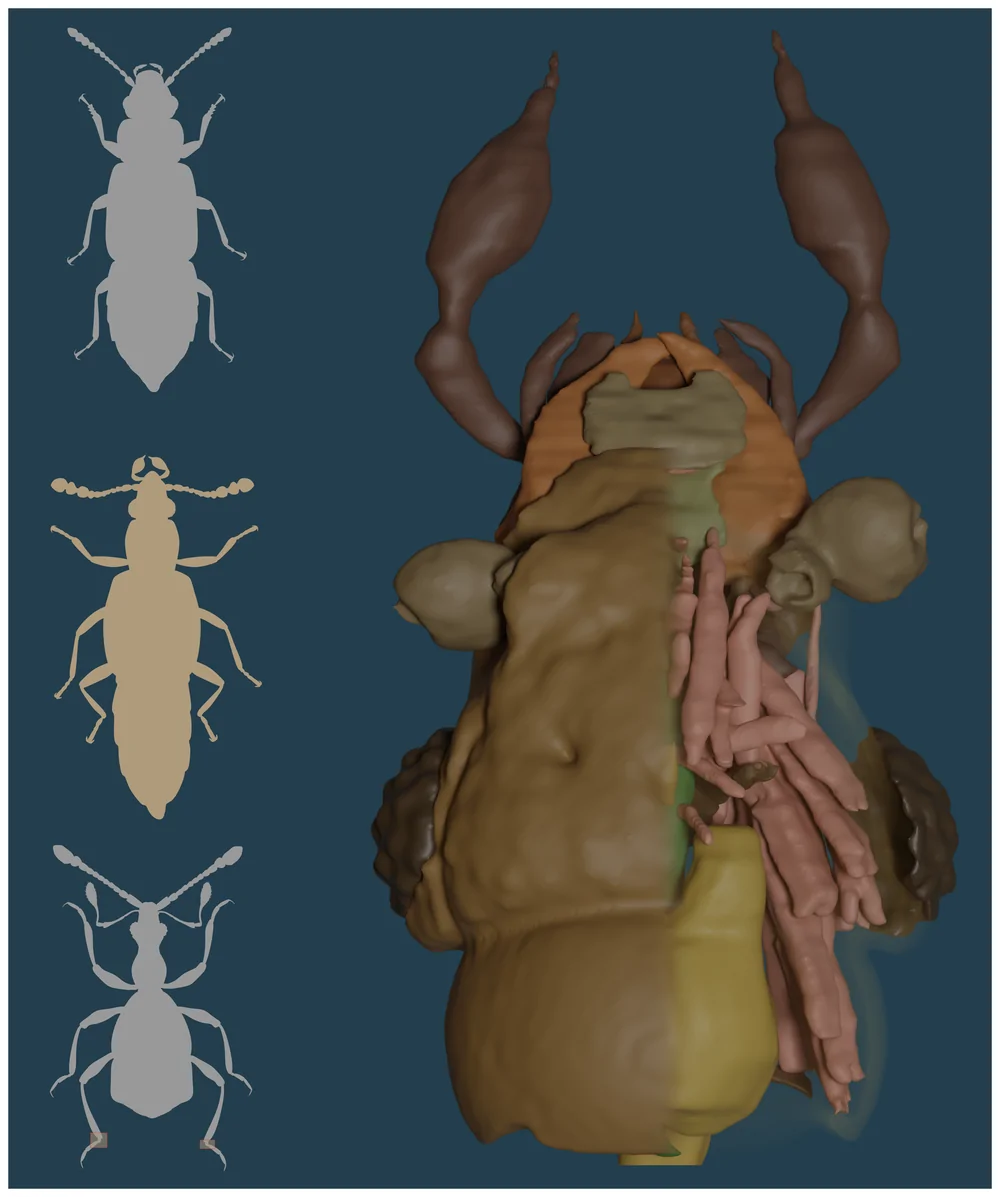

## 1 Introduction

The discovery of *Protopselaphus* Newton & Thayer and the introduction of Protopselaphinae significantly changed the systematics of Staphylinidae, by including the previously separate large family Pselaphidae as a subfamily (Newton and Thayer 1995). Moreover, Protopselaphinae and Pselaphinae were placed in a broad taxonomic context by a phylogenetic reconstruction as sister groups within the informal Omaliine Group, composed also of Dasycerinae, Empelinae, Glypholomatinae, Micropeplinae, Microsilphinae, Neophoninae, Omaliinae, and Proteininae (Newton and Thayer 1995). However, it must be noted that only three other (i.e., non‐Omaliine) subfamilies of Staphylinidae were included in the analysis, and since then, only the study of Yin et al. (2021) was also focused on this lineage with inclusion of *Protopselaphus*, but with even more limited selection of the outgroups, composed solely of one genus of the remotely related Osoriinae. Modern molecular analyses questioned the monophyly of the Omaline Group but corroborated close relationships of Pselaphinae, Dasycerinae and Neophoninae (McKenna et al. 2015). However, Protopselaphinae were not included. Moreover, a discovery of Cretaceous fossils of Dasycerinae that prompted a partial phylogenetic analysis of subfamilies potentially closely related to Pselaphinae (Yin et al. 2021) did not contribute to solving this issue, because of a strongly limited outgroup selection. The larvae of *Protopselaphus* may shed more light on relationships of this genus, but unfortunately, they remain unknown, and molecular data for Protopselaphinae are still lacking (Gusarov 2018; McKenna et al. 2015). Recently, not only the relationship of Protopselaphinae + Pselaphinae, but also the placement of Pselaphinae within the Omaliine Group was critically discussed by Kurbatov (2024), albeit without presenting a concurrent phylogenetic scenario tested by formal phylogenetic methods. Although only based on an informal character discussion, this critique is interesting because it may suggest a greater morphological (and possibly taxonomic) diversity of the extant Protopselaphinae. One of the characters indicated as incorrectly scored by Newton and Thayer (1995) is the transverse antebasal impression (i.e., the antebasal sulcus) on the pronotum, claimed by Kurbatov (2024) to be “completely absent” in *Protopselaphus*. This statement is supported by a scanning electron micrograph showing a specimen identified as *Protopselaphus* lacking the transverse sulcus. In contrast, the illustrations in Newton and Thayer (1995: figs. 1, 15, 35) clearly show this sulcus as present in the genus. As the presence and absence of sulci is commonly used in Pselaphinae as a genus‐level character, this finding suggests that a comprehensive study of the character diversity and composition of Protopselaphinae is important. This also underlines the necessity of more profound morphological studies of species of the subfamily in the context of the large and diverse Omaliine Group of rove beetles.

**Figure 1:**
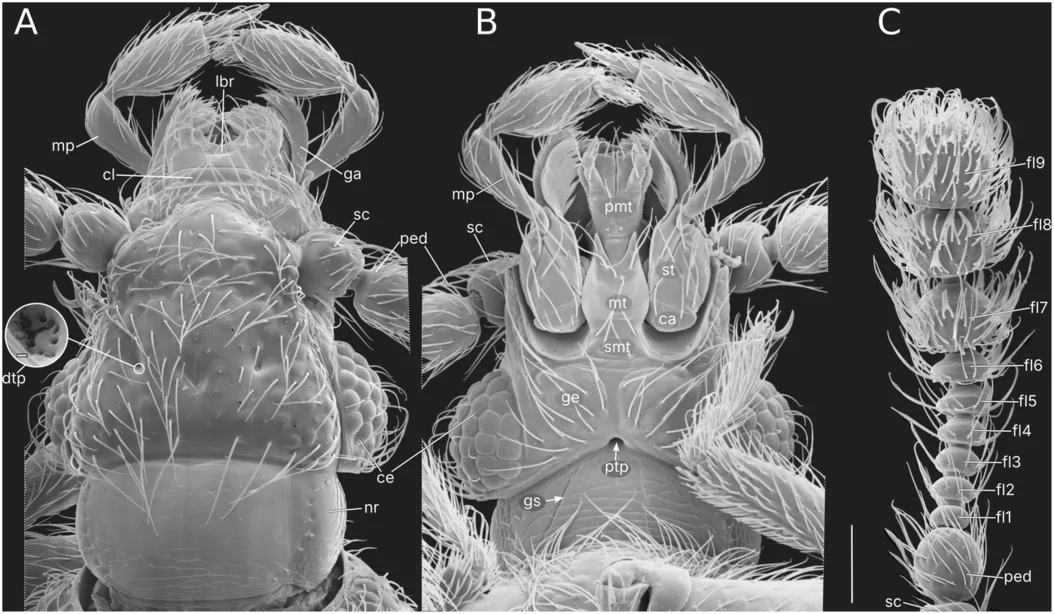
Head of *Protopselaphus* sp. from Thailand, SEM micrographs. (A) head, dorsal view; (B) head, ventral view; (C) right antenna, ventral view. ca, cardo; ce, compound eye; cl, clypeus; dtp, dorsal tentorial pit; fl1–9, flagellomeres 1–9; ga, galea; ge, gena; gs, gular sulcus; lbr, labrum; lc, lacinia; mp, maxillary palp; mt, mentum; nr, neck region; ped, pedicellus; pmt, prementum; ptp, posterior tentorial pits; sc, scapus; smt, submentum; st, stipes. Scale: A–C, 50 μm; D, 1 μm.

The known diversity of Protopselaphinae is low, but at the same time it represents a group of high phylogenetic significance. The eight extant nominal species are distributed in the peninsular Malaysia and Sarawak (undescribed species are known from China, Thailand, Laos and Vietnam (Nomura 2000; Nomura et al. 2008; Nomura 2011) and unpublished data), and few fossil representatives have been discovered in mid‐Cretaceous Myanmar amber (Liu et al. 2020, 2021). If this subfamily is indeed the sister group of Pselaphinae, its morphology is crucial for reconstructing the evolution of ant‐like litter beetles. An accelerated structural diversification has apparently taken place in this species‐rich subfamily (Parker 2016), resulting in a plethora of unique and bizarre forms. This could arguably be the background of the capacity to adapt to a very broad spectrum of lifestyles, reaching from predation in leaf litter specialized on Collembola (Schomann et al. 2008) to subterranean habits (Luo et al. 2021a, b), and last but not least close associations with ants (Parker and Grimaldi 2014). Jałoszyński et al. (2022) have shown how the information on external and internal cephalic structures of pselaphines can be crucial for understanding the phylogeny and diversification in this group. Obviously, this also applies to the phylogenetically crucial Protopselaphinae, a group treated comprehensively in Newton and Thayer (1995) but without anatomical data. This inspired us to fill in this important gap of information, by providing a detailed documentation of the morphology of the head of *Protopselaphus*, including SEM images showing details of the cuticular surfaces, but especially focusing on internal soft parts such as muscles, nervous system, glands, and elements of the anterior digestive tract. Material of *Protopselaphus*, especially preserved in suitable fixation fluids, is apparently very rare if available at all. The study we carried out was based on a single specimen stored in 70% ethanol, with sufficiently preserved internal soft parts for a detailed anatomical reconstruction. A list of characters with potential phylogenetic relevance is presented, with a main focus on anatomical features. At this point, we present no formal phylogenetic evaluation of our data set, which is limited to cephalic features. This will be carried out when detailed data are available for different body regions and a representative taxon sampling of pselaphine genera and suitable outgroup taxa.

## 2 Materials and Methods

### 2.1 Studied Species

#### 2.1.1 *Protopselaphus* sp. From Thailand, Undescribed

Single male specimen, collected in 2006 in Doi Inthanon N. P., Thailand, at 1600 m altitude by SN and preserved in 70% ethanol.

The specimen of *Dasycerus sulcatus* Brogniart was collected by Marek Wanat in 2021 in Switzerland in Sihlwald by sifting and preserved in 70% ethanol. *Tyrus mucronatus* Panzer was collected by Mateusz Sapieja in 2024 in Poland near the Białowieża Forest by sifting and preserved in 70% ethanol.

### 2.2 Synchrotron‐Radiation Micro‐Computed Tomography (SRµCT)

The examined specimen was transferred to 100% ethanol and placed in a pipette tip filled with the same liquid. The upper end of the tip was closed with hot glue, the lower end by melting. The sample was then scanned with a stable energy beam of 18 keV, 1.25 µm pixel size at the DESY Research Centre in Hamburg, Germany (Deutsches Elektronen Synchrotron, beamline PETRA III/IBL P05, beamtime scans performed under guidance of Dr. J. Hammel).

### 2.3 Computer‐Based 3D‐Reconstruction

The obtained image stack was cropped and adjusted with Fiji (= Image J). 3D Slicer 5.10 was used for the segmentation. The head capsule, compound eyes, antennae and tentorium were presegmented manually in 3D Slicer and subsequently semi‐automatically segmented using Biomedisa (Lösel et al. 2020). “Paint” and “Fill between slices” tools in 3D slicer were used to segment and reconstruct the mouthparts, musculature, nervous system and glands. All the reconstructed structures were exported as .stl files and imported in Blender 4.5.8, where the final editing and rendering was performed.

### 2.4 Scanning Electron Microscopy (SEM)

After the µCT data were obtained, the specimen was cleaned in a 10% aqueous solution of KOH for 1 h, washed in glacial acetic acid and distilled water (Schneeberg et al. 2017), dehydrated in an ascending ethanol series, transferred to 100% acetone and air dried. The head and prothorax were detached from the rest of the body, mounted on a Minutien pin, coated with gold (EmitechK 500X), and mounted on a rotatable specimen holder. Images of the head were taken with an SEM Zeiss EVO LS 10.

### 2.5 Image Plates

GIMP 3.2 (GPL license) was used to edit the SEM micrographs. Inkscape 1.4.3 (GPL license) was used for arranging and labelling the figure plates.

### 2.6 Terminology

The morphological nomenclature follows recent morphological studies on different body regions of Pselaphinae (Beutel et al. 2021; Jałoszyński et al. 2022; Luo et al. 2021a). For the musculature, the nomenclature of v. Kéler (1963) is used.

## 3 Results

### 3.1 Head Capsule

The moderately flattened head is prognathous, horizontally oriented, with the mouthparts anteriorly directed, aligned with the cephalic longitudinal axis (Figures 1A,B and 3). The head capsule is distinctly retracted into the prothorax posteriorly, with the hind margin of the compound eyes almost reaching the anterior pronotal margin. A moderately distinct, subcylindrical neck region (nr; Figure 1A,B) is present, twice as wide as long, with evenly rounded lateral margins, largely concealed within the anterior prothoracic region; it articulates with the prothorax in a condyle‐like manner but is only slightly constricted posterior to the compound eyes; its dorsal surface is demarcated from the dorsum of the anterior head region by a shallow sulcus. The compound eyes (ce; Figures 1A,B and 3) are strongly convex, semiglobular, very distinctly protruding, consisting of ca. 38 ommatidia with distinctly convex cornea lenses, with more than 15 setae of medium length inserted between them; the posterior surface is covered by a cuticular extension of the posterior genal area. In lateral view, the center of the compound eye is situated clearly below middle height of the head (Figure 3C). Ocelli are absent. The anterior cephalic region, anterior to the neck region and between the compound eyes, is moderately elongated, and its upper portion slightly narrowing anteriorly towards the antennal insertion area. The lateral margins of the preocular head region are nearly parallel over most of their length, but then very distinctly, obliquely converging towards the clypeus (Figure 1A). The dorsal side of the head bears distinctly visible small and asetose dorsal tentorial pits, a shallow median unpaired pit slightly posterad the dorsal tentorial pits, and a narrow longitudinal supraocular sulcus along the dorsomesal margin of each compound eye, clearly demarcating them from the frontovertexal region (Figure 1A). The dorsal tentorial pits (dtp; Figure 1A) resemble glandular porous fields, that is, each bears an irregular ring of minute pores at its bottom; the median pit is directed ventroposterad and its lumen is not visible in dorsal view. The anteromedian margin of the frontal region is almost straight, only very slightly convex. The clypeus (cl; Figure 1A) is short and transverse, and only slightly sloping anteroventrad in lateral view; anterolaterally it is evenly rounded and the anterior margin is only very slightly convex; the clypeofrontal border is marked by a low transverse ridge. The antennal insertion area is located anterolaterally, but distinctly posterior to the anterior frontal margin; the antennal insertion is located dorsolaterally and is covered by an anterolateral flat projection of the elevated portion of the anterior frontal region, thus the antennal fossa is not visible from above. The ventral surface of the head capsule is slightly shorter than the dorsal side posteriorly, and more distinctly shorter anteriorly (Figure 3C). The neck region is demarcated ventrally by an anteriorly convex transverse sulcus (a ventral extension of the occipital constriction), which medially connects with an unpaired deep and slightly oval posterior tentorial pit. External vestiges of the gular sulcus (gs; Figure 1B) are present in form of two posterolaterally diverging faint fissures (only visible in SEM micrographs). Anteriorly the gular region is fused with the submental area, which is in turn completely fused with the anterolateral wall of the head capsule.

The dorsal area of the anterior head region is covered with a fairly dense vestiture of long setae (ca. 0.5–0.8 mm) inserted between weakly elevated and diffuse tubercles (ca. 8 µm in diameter). In contrast, the dorsal surface of the neck is mostly glabrous, except for a posterior microreticulation composed of irregular, transverse polygonal cells; the microsculpture extends onto the entire lateral and ventral sides of the neck (Figure 1A,B). Two curved regular rows of eight short (ca. 0.1 mm) laterally oriented setae, probably mechanoreceptors, extend from the posterodorsal neck region to its anterior margin, close and parallel to the lateral margin (Figure 1A). On the ventral side of the neck three short setae (ca. 0.1 mm) are present laterad the gular sulci (Figure 1B).

### 3.2 Cephalic Endoskeletal Structures

A thin, curved tentorial bridge is present at the ventral postoccipital foramen, completely separated from the rest of the tentorium (Figure 5C). Internal gular ridges are entirely missing. The posterior and dorsal arms form nearly vertical columns, which are apically firmly fused with the head, forming distinct dorsal tentorial pits (Figures 4B and 5E). The flattened posterior tentorial arms diverge above the slightly elevated shared base (Figure 5E) that arises from a very distinct unpaired ventral pit (ptp; Figure 1B). Long and distinctly developed anterior tentorial arms are present; their dorsoventrally flattened posterior sections have a parallel orientation, in contrast to the rest of the arms that diverge anterolaterally (Figure 5C). The anterior tentorial pits are lacking. The dorsal and anterior tentorial arms are approximately equally thick (Figure 5E).

### 3.3 Labrum

**Figure 2:**
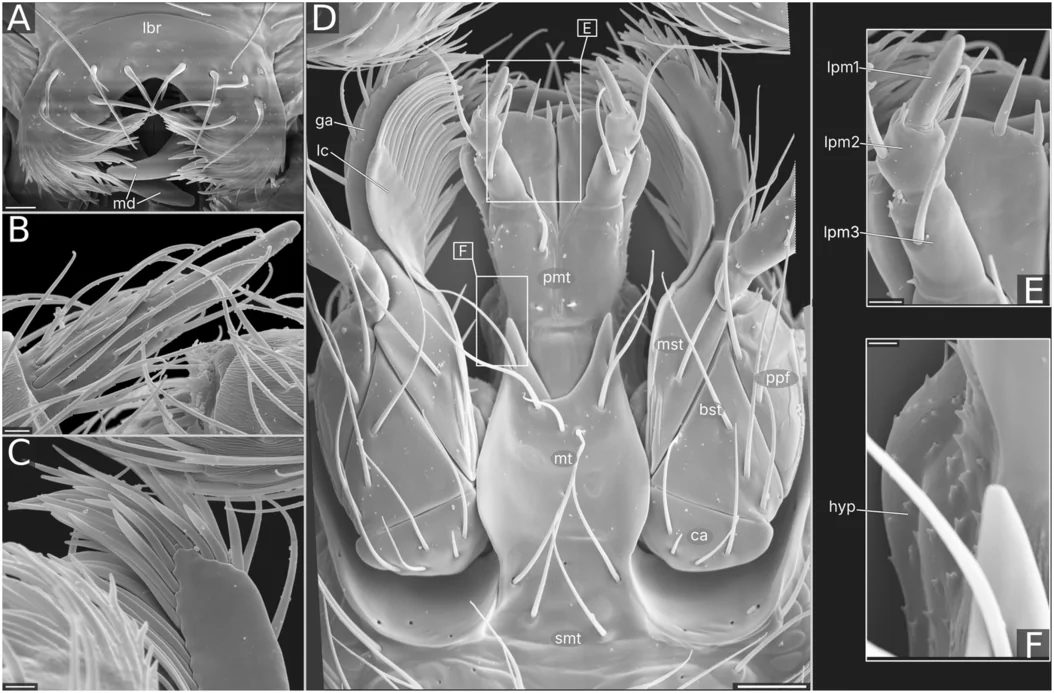
Mouthparts of *Protopselaphus* sp. from Thailand, SEM micrographs. (A) labrum, anterodorsal view; (B) left maxillary palpomere 4, dorsal view; (C) right lacinia, dorsal view; (D) mouthparts (maxillary palps cropped), ventral view; (E) labial palp, ventral view; (F) lateral wall of the anterior hypopharynx. bst, basistipes; ca, cardo; ga, galea; hyp, hypopharynx; lbr, labrum; lc, lacinia; lpm1–3, labial palpomeres 1–3; md, mandible; mst, mesostipes; mt, mentum; pmt, prementum; ppf, palpifer; smt, submentum. Scale: A: 10 μm, B: 4 μm, C: 4 μm, D: 20 μm, E: 5 μm, F: 2 μm.

**Figure 3:**
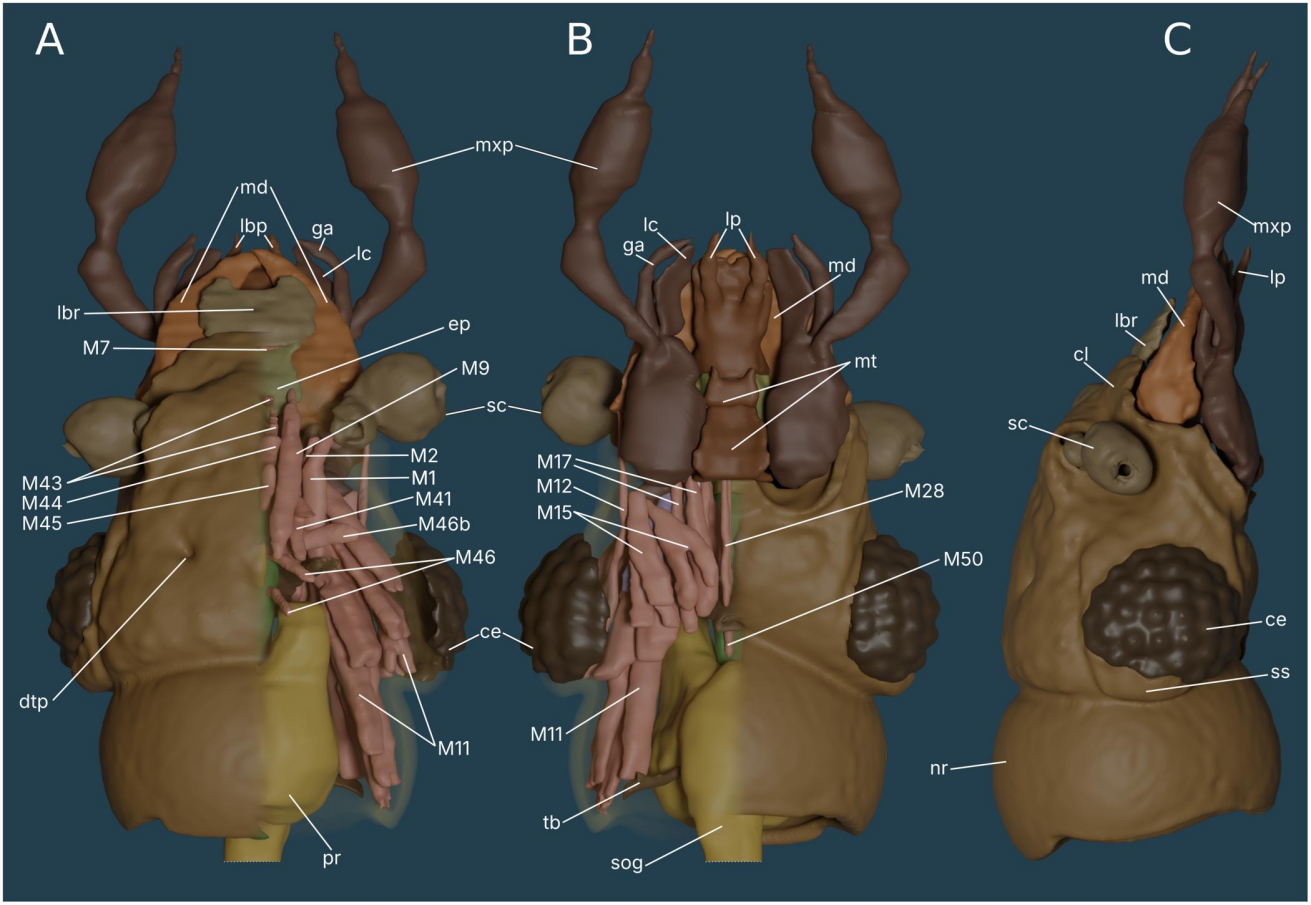
Head of *Protopselaphus* sp. from Thailand, 3D reconstruction. (A) head, dorsal view, right side of the head capsule semi‐transparent; (B) head, ventral view, left side of the head capsule semi‐transparent; (C) head, lateral view. ca, cardo; ce, compound eye; cl, clypeus; dtp, dorsal tentorial pit; ep, epipharynx; fl, flagellum; ga, galea; lbp, labial palp; lbr, labrum; lc, lacinia; li, ligula; lp, labial palp; md, mandible; mt, mentum; mxp, maxillary palp; M1, M. tentorioscapalis anterior; M2, M. tentorioscapalis posterior; M7, M. labroepipharyngalis; M11, M. craniomandibularis internus; M12, M. craniomandibularis externus; M15, M. craniocardinalis; M17, tentoriocardinalis; M 28, M. submentopraementalis; M 41, M. frontohypopharyngalis; M43, M. clypeopalatalis; M44, M. clypeobuccalis; M45, M. frontobuccalis anterior; M46, M. frontobuccalis posterior; M50, M. tentoriobuccalis posterior; nr, neck region; ped, pedicellus; pr, protocerebrum; sc, scapus; sog; suboesophageal ganglion; ss, supraocular sulcus.

The labrum is present as a transverse plate‐like structure (Figures 1A and 2A), connected with the clypeus by an internal membranous fold (Figure 3). The anterolateral edges are rounded, separated by a broad median emargination. Twelve long (ca. 35 µm), very distinct setae are present on the dorsal surface, mostly close to the anterior margin, as well as two short setae (ca. 8 µm) at the anterior edge close to the median line. A very dense comb of long (ca. 30 µm; almost as long as the labrum) antero‐medially oriented microtrichia is present along the anterior labral margin laterad the medial emargination. The anterolateral margins of the median emargination bear two pairs of minute peg‐like sensilla directed anteromesad; a pair of sensilla, presumably sensilla campaniformia, is situated in the median region of the labrum, behind the median pair of setae (Figure 2A). A pair of minute lateral pores, probably glandular openings, is present near the posterior labral margin (one of them is visible on the left side of Figure 2A). The tormae are not discernible in the µCT scans.

**Figure 4:**
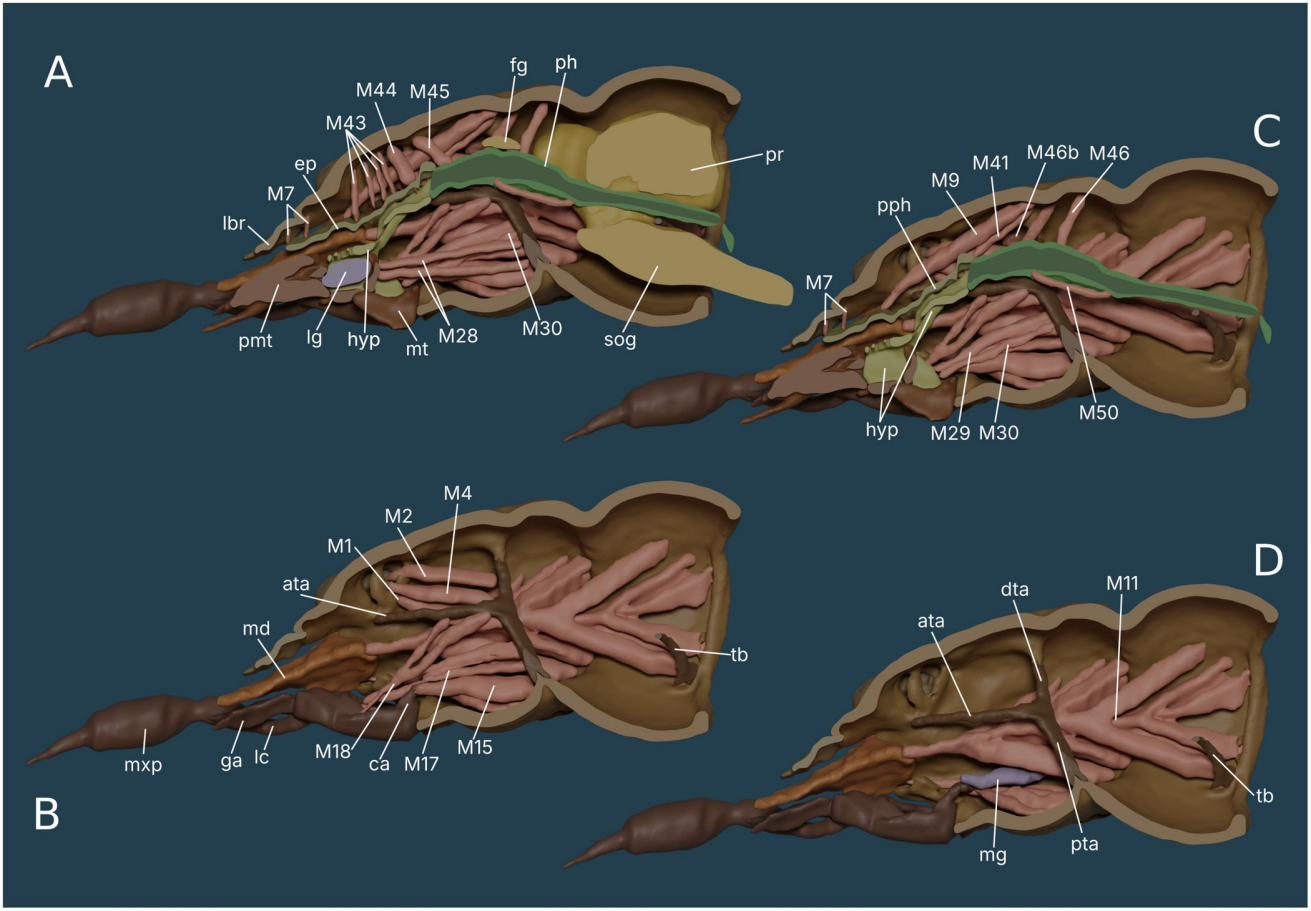
Head of *Protopselaphus* sp. from Thailand, 3D reconstruction, sagittal section with selected structures blended out in B–D. ata, anterior tentorial arm; ca, cardo; dta, dorsal tentorial arm; ep, epipharynx; fg, frontal ganglion; ga, galea; hyp, hypopharynx; lbr, labrum; lc, lacinia; lg, labio‐hypopharingeal gland; mg, maxillary gland; mt, mentum; mxp, maxillary palp; ph, pharynx; pmt, prementum; pph, prepharynx; pr, protocerebrum; pta, posterior tentorial arm; sog, suboesophageal ganglion; tb, tentorial bridge.

Musculature (Figure 4C): M7, M. labroepipharyngalis, two thin but distinct paired bundles, Origin (O): dorsal wall of the labrum, close to the median line, Insertion (I): anterior area of the epipharynx; M9, M. frontoepipharyngalis, long and strongly developed muscle, O: frontal area, close to the midline, directly anterior to the mesal pit, I: laterally at the posterior corners of the ventral wall of the labrum, that is, the anterior epipharynx.

### 3.4 Antennae

The antenna is composed of 11 segments. The scapus (sc; Figures 1 and 3) is inserted below the anterolateral frontal projection with a small articulatory basal piece, distinctly curved dorsad, and partly visible in frontal view; the main distal part of the scapus is barrel‐shaped. The subglobular pedicellus (ped; Figure 1C) is distinctly narrowed proximally, about as long as the scapus, and about as wide distally. Flagellomeres 1–8 appear almost disc‐shaped, with a very distinct proximal peduncle; their width distinctly increases distad, but their length only slightly; antennomere 3 is much narrower and shorter than the pedicellus. A distinct, even though loose club is formed by antennomeres 9–11, which are much larger than the proximal flagellomeres; these subglobular distal flagellomeres also bear a distinct proximal peduncle, which articulates with the distal surface of the preceding segments. A dense vestiture of two distinctive types of setae is present on the surface of the antennae; thicker and curved setae are concentrated on the antennomeres forming the club; otherwise, the entire flagellum is covered with numerous long (ca. 35–40 µm) and some medium‐length (ca. 20–25 µm) and thin setae (Figure 1C).

Musculature (Figure 4B): M1, M. tentorioscapalis anterior, O: anteriormost area of the posterior tentorial arm and ventral area of the base of the anterior arm; I: not clearly visible in the data set, likely the anteroventral area of the articulatory membrane of the scapus; M2, M. tentorioscapalis posterior, O: upper region of the dorsal tentorial arm, I: with a short tendon dorsally on the posterior margin of the scapal base; M4, M. tentorioscapalis medialis, O: basal region of the dorsal tentorial arm, directly above the origin of the anterior arm and between the areas of origin of M1 and M2, I: medioventrally on the articulatory membrane of the scapus. The three extrinsic antennal muscles are arranged parallel to each other. M5 and M6, M. scapopedicellaris externus and internus, both are present but the exact sites of origin and insertion are not recognisable in the µCT data.

### 3.5 Mandibles

**Figure 5:**
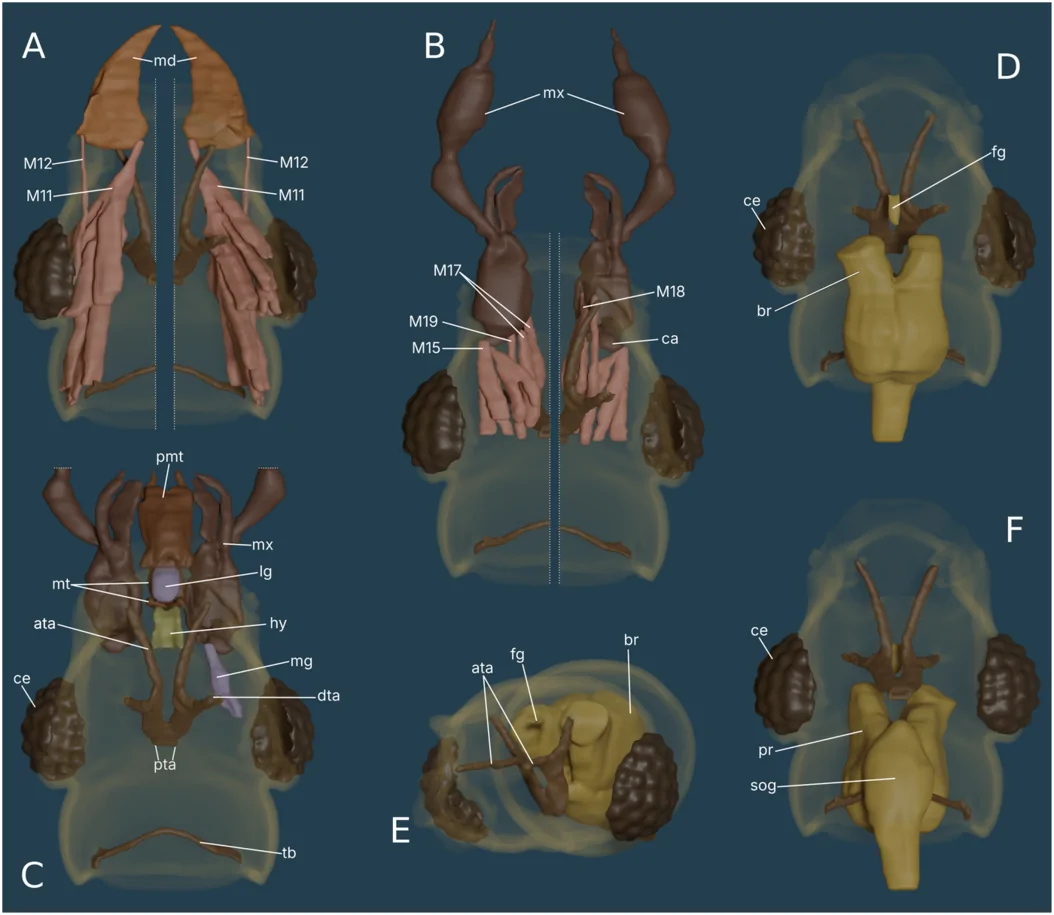
Head of *Protopselaphus* sp. from Thailand, 3D reconstruction, musculature (A and B), glands (C) and nervous system (D–F); head capsule semi‐transparent. (A) mandibular muscles, dorsal (right) and ventral (left) view; (B) maxillary muscles, dorsal (right) and ventral (left) view; (C) cephalic glandular system and tentorium, dorsal view; (D) nervous system, dorsal view; (E) nervous system, anterolateral view; (F) nervous system, ventral view. ata, anterior tentorial arm; br, brain; ca, cardo; ce, compound eye; dta, dorsal tentorial arm; fg, frontal ganglion; hy, hypopharynx; lg, labio‐hypopharyngeal gland; md, mandible; mg, maxillary gland; mt, mentum; mx, maxilla; pmt, prementum; pr, protocerebrum; pta, posterior tentorial arms; sog, suboesophageal ganglion; tb, tentorial bridge.

The strongly sclerotized mandibles (md; Figure 5A) are triangular, only slightly curved distally, with a relatively wide basal portion; the distinctly narrowed, slender distal regions are intercrossing medially in the resting position (Figure 2A). The mesal mandibular bases are distinctly separated from each other; the molar area is slightly convex but a grinding surface is absent; subapical teeth are lacking. Several long setae are present along the lateral mandibular margin of *Protopselaphus watrousi* (Newton and Thayer 1995: fig. 8) and also a long and dense dorsomesal brush of microtrichia (“prostheca”). We could not visualize these structures with the single specimen at hand.

Musculature (Figure 5A): M11, M. craniomandibularis internus, largest cephalic muscle, consisting of two components that share a strong tendon, O: large area of the lateral wall of the head capsule, M11a anterad the occipital constriction, M11b posterolateral area of the neck region; I: mesal mandibular base with the tendon; M12, M. craniomandibularis externus, distinctly smaller than M11, consisting of four rather short bundles with a shared long and thin tendon, O: lateral area of the head capsule anterad the neck region, between bundles of M11; I: with the tendon on the lateral mandibular base.

### 3.6 Maxillae

The maxillae are inserted in the maxillary fossae laterad the anteromedian portion of the submental region and the posterior region of the mentum; the surface of the fossa is smooth and sclerotized and bears three sensilla (Figures 1B and 2D). The cardo (ca; Figure 4D) is very short and transverse, with an articulatory process ascending from the posterodorsal margin. Five (only four visible on the left cardo) setae are inserted on the posteroventral surface of the cardo (Figure 2D). The stipes is about twice as long as wide and separated from the cardo by a transverse sulcus. Three long (40–50 µm) setae are inserted close to the lateral margin of the basistipes, and a single seta is inserted on the mediostipes. A narrow membranous area is visible between the mediostipes and the mentum. The palpifer (ppf; Figure 2D), located laterad the basistipes, is distinctly elongated and of conical shape; it bears two long ventral setae close to its distomesal margin. Both endite lobes are connected to the apical region of the mediostipes; a dense fringe of curved setae is visible on the distomesal margin of the galea (Figure 2C), several long setae are inserted close to the lateral margin, and a group of minute tubercle‐like structures is present on the dorsal side of the basal area, possibly sensilla; the long and curved lacinia also bears a dense fringe of curved setae (ca. 30 µm) at its apical margin (Figure 2D). The palp is large compared to the other mouthparts including the maxillary body. The short, subcylindrical palpomere 1 is inserted between the apical region of the palpifer and a distinct notch of the distal mediostipes; it is distinctly bent laterad; palpomere 2 is about 3 times as long, distinctly curved, and very distinctly widened distally; palpomere 3 is the largest, slightly longer but much more voluminous than palpomere 2, strongly widening distally; the apical palpomere 4 is subconical, about 1/2 as long as 3, slender, narrowing distally and apically rounded (Figure 1A,B). Palpomere 1 is glabrous, bearing two campaniform sensilla on its ventral surface, whereas palpomeres 2–4 are evenly covered with long setae (ca. 25–20 µm), except for the distal asetose half of palpomere 4; three strongly developed, flattened and elongate sensilla (2 dorsally and 1 ventrally; ca. 20 µm long) are inserted on the proximal region of palpomere 4. The cuticular surface of palpomeres 1–2 and 4 is mostly smooth, whereas palpomere 3 shows a pattern of longitudinal parallel grooves with some degree of dichotomy and anastomosis.

Musculature (Figures 4B and 5B): M15, M. craniocardinalis, large muscle, composed of two components with one insertion, O: broad area of the ventral wall of the head capsule, not reaching the neck region, M15a laterad M15b, I: posterolateral edge of the lateral branch of the cardinal process; M17, M. tentoriocardinalis, three thick separate bundles, O: ventral half of the posterior tentorial arm and ventral wall of the head capsule laterad their base, I: separately on the mesal branch of the cardinal process and membranous area above it; M18, M. tentoriostipitalis, two bundles with distinctly separated origins merging on one thin tendon, O: proximal and mid‐area of the anterior tentorial arm, I: with a tendon dorsally on the mediostipital base; M19, M. craniolacinialis, long, circular in cross‐section and with a constant width over its length, O: laterad the ventral tentorial base, I: base of lacinia. The intrinsic muscles are not clearly recognisable in the data set.

### 3.7 Labium

The anterior region of the submentum, inserted behind the cardines, is subrectangular, steep and relatively narrow, with slightly concave lateral margins and bearing four long setae; a slightly recessed and asetose median region, rather narrow over most of its length but widening anteriorly, is possibly an exposed posterior submental region, posteriorly meeting the unpaired posterior tentorial pit; the posterior submentum (smt; Figure 1B) is apparently largely displaced by medially directed genal extensions. The mentum (mt; Figure 2D) is evenly rounded laterally in its proximal region, slightly narrowed towards its posterior margin, and also narrowing anteriorly, ending with two triangular and apically pointed anterolateral processes, separated by a deep median emargination; the ventral surface bears three distolateral pairs of long setae. The basal part of the prementum appears unpigmented and is apparently unsclerotized; a pair of small sensilla, inserted relatively close to the midline, is present anterior to this region. The labial palps are inserted on an indistinct flat and broad palpifer at the base of the medially adjacent hypopharyngeal superlinguae, which together form a broad, flat structure, with a smooth and glabrous ventral surface; a pair of short setae is inserted at the anterior edge of the superlinguae, immediately close to the median line, and a longer pair more laterally; a dense fringe of microtrichia on the apical margin is missing (Figure 2D). The labial palp is three‐segmented and much smaller than the maxillary palp; the cylindrical palpomere 1 is the longest and bears one long ventral seta inserted in the middle section, as well as a short stout seta laterally at the base; palpomere 2 is ½ as long as palpomere 1, slightly widened near its middle section and also bears a long seta; at its lateral apical margin it bears a pair of very short, spine‐like sensilla. The apical palpomere is elongate, slender, gradually tapering from the base to the blunt apex, almost as long as palpomere 1 and asetose (Figure 2E).

Musculature (Figures 3B and 4A): M28, M. submentopraementalis, two separate paired bundles with adjacent sites of origin and insertion, O: ventral base of the tentorium, I: dorsomedially on the hind margin of the prementum; M29, M. tentoriopraementalis inferior, O: posterior tentorial arm dorsad M 28, I: posterolaterally on the lateral wall of the anterior hypopharynx; M30, M. tentoriopraementalis superior, O: posterior tentorial arm, ventrad M29, I: posterolaterally on the anterior hypopharynx p; M34, M. praementopalpalis, not visible.

### 3.8 Epipharynx and Hypopharynx

The epipharynx (ep; Figure 4A) is divided into two distinct areas: the rather broad anterior epipharynx, equivalent with the ventral wall of the labrum, and a distinctly narrower posterior epipharyngeal region that forms the cibarial roof and the dorsal wall of the prepharyngeal tube (pph, Figure 4A,C); both epipharyngeal parts appear distinctly sclerotized. The ventral surface of the anterior epipharynx apparently bears a median fringe of microtrichia (only indistinctly visible in the data set). The anterior hypopharynx (hyp; Figure 4C) is fused with the anterior labium; only the lateral walls are visible externally, sparsely but evenly covered with short microtrichia. The posterior hypopharynx, laterally fused with the posterior epipharynx, forms the prepharyngeal floor (Figure 4A,C); it rises rather steeply towards the anatomical mouth opening, and is enclosed by distinctly sclerotized hypopharyngeal suspensorial arms.

Musculature (Figure 4A): M41, M. frontohypopharyngalis, well‐developed muscle, two distinct bundles merging onto one long, strongly developed tendon, O: central frontal region, laterad M9 I: apex of suspensorial arms at posterior edge of the prepharyngeal tube, laterad the anatomical mouth; M43, M. clypeopalatalis, at least four thin paired bundles, O: frontal region close to the median line; I: epipharyngeal wall of the prepharyngeal tube; M44, M. clypeopalatalis, a single pair of strong bundles between the last bundle of the posterior subunit of M43 and M45; O: I: dorsal wall of the prepharyngeal tube, directly behind the anatomical mouth opening; MmIII, M. buccalis transversalis, a strong transverse bundle anterior to the anatomical mouth.

### 3.9 Pharynx

The pharyngeal tube (ph; Figure 4A) is fairly wide anteriorly but distinctly narrowed and round in cross section in the neck region.

Musculature (Figure 4A,C): M45, M. frontobuccalis anterior, single paired bundle, O: frontal area posterad M44, I: dorsally on the anteriormost pharynx, directly behind the anatomical mouth opening; M46, M. frontobuccalis posterior, two components, M46a: two long oblique bundles, O: frontal region, posterior to M41, I: dorsal pharyngeal wall, anterior to the brain, M46b: strongly developed, almost horizontal paired bundle, O: frontal region, directly posterad the connection of the dorsal tentorial arms with the head capsule, I: laterodorsally on the anterior pharynx, directly anterad M46a. M48, absent. M50, M. tentoriobuccalis posterior, a pair of distinct bundles extending parallel to the ventral wall of the anterior pharynx, O: ventrally on the pharyngeal wall, I: ventrally on the anterior pharynx, directly below the insertion of M46a.

### 3.10 Nervous System

The brain, that is, the supra‐ and suboesophageal ganglionic complexes, are shifted to the neck region, which they fill out almost completely. The morphologically anterior portion of the two protocerebral hemispheres extends into the postoccipital region of the posterior head capsule. The large, oval suboesophageal complex (sog; Figure 5F) fills out the ventral half of the neck region. The compact frontal ganglion (fg; Figure 4A) is placed parallel to the anterior pharynx between the two paired bundles of M46a.

### 3.11 Glands

Two distinct cephalic clusters of glands are present: an unpaired labio‐hypopharyngeal gland and paired maxillary glands (Figure 5C). The former lies in the space above the anterior mentum and is laterally constricted by the walls of the anterior hypopharynx (mg; Figures 4A and 5C). The maxillary glands are located posterad the cardinal process and dorsad M15 and extend lateroposterad, almost reaching the lateral wall of the head capsule (Figure 4D). Additionally, diffuse tissue is visible in the mandibular lumen and in the area posterad the mandibular bases, possibly mandibular glands. However, a precise identification is not possible without histological sections.

## 4 List of Potential Phylogenetically Relevant Characters

### 4.1 Characters of the Head

**Figure 6:**
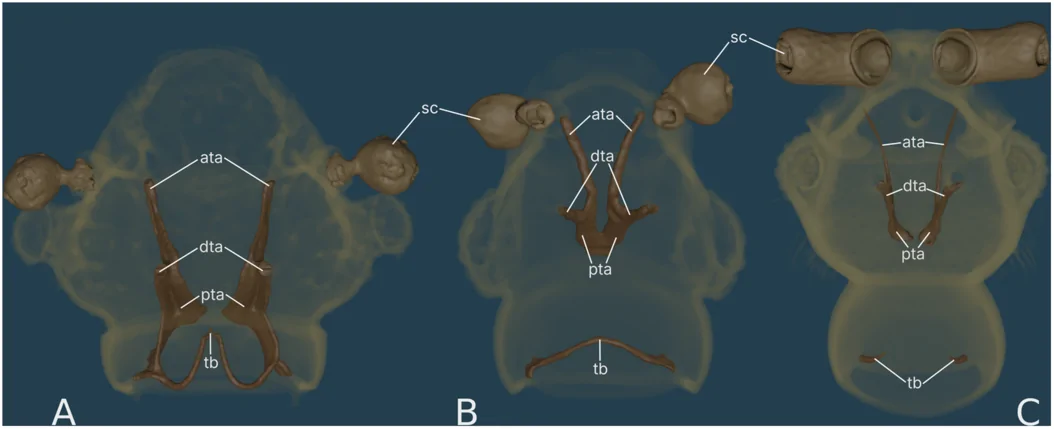
Comparison of antennal insertion and tentorium structure in *Dasycerus sulcatus* (A), *Protopselaphus* sp. from Thailand (B) and a “higher” pselaphine *Tyrus mucronatus* (C); 3D reconstruction of the head capsule (rendered half‐transparent), tentorium and scapus, dorsal view. ata, anterior tentorial arms; dta, dorsal tentorial arms; pta, posterior tentorial arms; sc, scapus; tb, tentorial bridge.

1. Foveae on dorsal side of the head (Newton and Thayer 1995: chars. 1, 2): (0) absent; (1) shallow concavity on the frontal region; (2) dorsal foveae present. Distinct dorsal foveae (other than tentorial pits) are absent in *Protopselaphus*, in extinct species of Dasycerinae (Yin et al. 2021), and also in most other groups of Staphylinidae with some exceptions (Micropeplinae partim, Dasycerinae partim) (Newton and Thayer 1995). An indistinct median concavity is visible in the specimen of *Protopselaphus* we examined (Figure 1A), but was not shown or mentioned in Newton and Thayer (1995) (dorsal foveae coded as absent in character state matrix). The presence of distinct foveae is a characteristic feature of Pselaphinae and arguably an autapomorphy. However, their distribution varies strongly (e.g., Chandler 2001) and it is difficult to assess a groundplan condition for the subfamily.
2. Neck region separated from occipital region of main part of head: (0) absent; (1) sub‐hemispherical, only slightly constricted in postocular region; (2) very distinct, hemispherical, strongly constricted. A distinctive, condyle‐like hemispherical neck region, as it is generally present in Pselaphinae (e.g., Beutel et al. 2021; Chandler 1991; Jałoszyński et al. 2020; Jeannel 1950: région collaire bien individualisée; Nomura 1991) is an autapomorphy of the subfamily. A rounded sub‐hemispherical postocular part of the head capsule of *Protopselaphus* is likely an intermediate condition. A hemispherical neck region is missing in most groups of Staphylinidae (except for many Scydmaeninae; Jałoszyński 2025) and very likely absent in the groundplan of the family (Blackwelder 1936: fig. 10; Thayer 2016; Thayer and Newton 1979: fig. 7). A very narrow neck region with subparallel lateral edges occurs in extinct species of Dasycerinae (Yin et al. 2021: fig. 4a,b,).
3. Placement of compound eye in lateral view: (0) central point of compound eye at or above half‐height of head capsule; (1) in ventral half of head capsule. Ventrally shifted eyes occur in most groups of higher Pselaphinae and in *Protopselaphus*. This condition is very rare among other groups of Staphylinidae.
4. Shape of clypeal region: (0) slightly declined; (1) steep. A slightly inclined clypeal region as it occurs in extant and extinct species of Dasycerinae and Protopselaphinae (Newton and Thayer 1995; Yin et al. 2021) and very rarely in Pselaphinae (e.g., *Bergrothia*; Luo et al. 2021a: figs. 1A and 4D,F), is a plesiomorphic condition. It is steep in *P. heisei* (Beutel et al. 2021) like in Clavigeritae (Jałoszyński et al. 2020) and various other groups of Pselaphinae including Faronitae (e.g., Park and Carlton 2015: fig. 3l,m). This is likely a groundplan apomorphy of Pselaphinae, with few reversals.
5. Antennal foramina: (0) not shifted to ventral side of supraantennal lobe; (1) shifted to ventral side of supraantennal lobe. An anterolateral insertion of the antenna as it occurs in extant and extinct Protopselaphinae (Newton and Thayer 1995) is a plesiomorphic condition and likely also part of the groundplan of Pselaphinae. A conspicuous derived feature that likely evolved within this subfamily is an insertion on the ventral side of the supraantennal lobe. This condition is documented for *P. heisei* (Beutel et al. 2021) and is also present in *Bryaxis* Kugelann (Jeannel 1950: fig. 1). This highly unusual apomorphic condition is not present in *Claviger* (Jałoszyński et al. 2020) and *Bergrothia* (Luo et al. 2021a), and is also missing in Faronitae and other groups of the subfamily.
6. Posterior tentorial pits (Chandler 2001: gular foveae): (0) separated; (1) connected. A slightly curved, undivided transverse fissure is present between the tentorial columns in *Protopselaphus* (Newton and Thayer 1995: fig. 3). The posterior tentorial grooves are also connected in *Bergrothia* (Luo et al. 2021a: figs. 1C and 4C) and many other pselaphines. This is an apomorphic feature shared by Protopselaphinae and many representatives of Pselaphinae. However, the condition varies strongly, for instance with posterior pits distinctly separated in species of *Faronus*, or also in *Pselaphus heisei* (Beutel et al. 2021). The posterior pits are almost always distinctly separated in other groups of Staphylinoidea (e.g., Antunes‐Carvalho et al. 2017; Blackwelder 1936) including *Dasycerus sulcatus* Brogniart, and also outside the superfamily (Anton and Beutel 2004; Dressler and Beutel 2010).
7. Tentorial bridge: (0) uninterrupted; (1) interrupted; (2) absent. The tentorial bridge is complete in *Protopselaphus* (Newton and Thayer 1995: Figs. 2 and 3) and in the groundplan of Staphyliniformia, Staphylinoidea and Staphylinidae (e.g., Anton and Beutel 2004; Antunes‐Carvalho et al. 2017; Beutel et al. 2003; Weide and Betz 2009: Fig. 5). It is present but interrupted in *P. heisei* (Beutel et al. 2021), whereas it is absent in *Bergrothia* (Luo et al. 2021a) like in *Claviger* and *Diartiger* (Jałoszyński et al. 2020, 2022). It is likely that the median interruption is an autapomorphy of Pselaphinae and that its loss occurred multiple times in the subfamily.
8. Location of tentorial bridge: (0) connecting paired elements of gular ridges distant from foramen occipitale; (1) located at foramen occipitale. The tentorium usually forms a continuous structural unit in Staphylinoidea, even though the bridge can be shifted backwards on the gular ridges to varying degrees (e.g., Blackwelder 1936: fig. 1F; Newton and Thayer 1995; Weide and Betz 2009). In contrast to the typical condition, the tentorial bridge is located at the foramen occipitale in *Protopselaphus* (Newton and Thayer 1995: figs. 2 and 3) and also in *P. heisei* (Beutel et al. 2021), distinctly separated from the dorsoventral main tentorial arms. This is a potential synapomorphy of Protopselaphinae and Pselaphinae (Newton and Thayer 1995). Various degrees of reductions of the bridge occur in pselaphines (e.g., Jałoszyński et al. 2020, 2022; Luo et al. 2021a).
9. Anterior tentorial arms: (0) distinctly developed; (1) very thin (“filament‐like”, Figure 6C); (2) or absent. Present and well‐developed in *Protopselaphus* (Newton and Thayer 1995: Figs. 2 and 3). Present but very thin in *Bergrothia* (Luo et al. 2021a: Figs. 4F and 6E), and also present but extremely thin in *P. heisei* and *Claviger* (Jałoszyński et al. 2020; Beutel et al. 2021). The anterior arms are usually distinctly developed in Staphylinidae (e.g., Newton and Thayer 1995; Weide and Betz 2009), for instance in *Dasycerus*, and also in most other groups of beetles. Thin anterior arms may be a groundplan apomorphy of Pselaphinae, with multiple complete reductions within the subfamily. The absence of muscle attachment sites on these structures is correlated with this condition.
10. Tentorial columns; (0) absent; (1) present. Massive vertical column‐like tentorial elements comprising the posterior and dorsal arms (Figure 6B,C) are present in *Protopselaphus* and Pselaphinae (Beutel et al. 2021; Jałoszyński et al. 2020, 2022; Luo et al. 2021a; Newton and Thayer 1995). A similar condition was found in one genus of Scydmaeninae (*Chevrolatia* Jacquelin du Val; Jałoszyński 2025) and one genus of Euaesthetinae (*Edaphus* Motschulsky; Jałoszyński, unpublished obs.). The posterior and dorsal arms do not form such compact units in other groups of Staphylinoidea and the dorsal arms are usually only attached by fibrillae if present (e.g., Antunes‐Carvalho et al. 2017; Weide and Betz 2009: fig. 5).
11. Dorsal tentorial pits: (0) absent; (1) present. Dorsal tentorial pits are present in *Protopselaphus* (Newton and Thayer 1995) and most species of Pselaphinae (Beutel et al. 2021; Jałoszyński et al. 2020, 2022; Luo et al. 2021a), but can be entirely reduced in some genera (e.g., some *Eupines* King; Chandler 2001). Pits in this position are also externally visible in *Dasycerus sulcatus*, as indicated by SEM images, but not recognizable in extinct species of the subfamily shown in Yin et al. (2021), and dorsal pits also occur in some Micropeplinae, Omaliinae, Neophoninae, and in *Chevrolatia* Jacqueline du Val (Scydmaeninae). Internally the pits are adjacent with the dorsal tentorial arms in *Dasycerus sulcatus* but without a fusion. The dorsal pits are absent in other groups of Staphyliniformia (Antunes‐Carvalho et al. 2017; Beutel et al. 2003; Blackwelder 1936: fig. 1; Jałoszyński 2025; Yavorskaya et al. 2023) but the presence in some groups of Staphylinidae cannot be excluded at present.
12. Laminatentoria: (0) present; (1) absent. Laminatentoria are missing in *Protopselaphus* (Newton and Thayer 1995: figs 2 and 3) and in all examined species of Pselaphinae (Beutel et al. 2021; Jałoszyński et al. 2020, 2022; Luo et al. 2021a), and also in *Dasycerus sulcatus*. A laminatentorium is likely present in the groundplan of Staphyliniformia, Staphylinoidea and Staphylinidae (e.g., Anton and Beutel 2004; Antunes‐Carvalho et al. 2017; Beutel et al. 2003; Blackwelder 1936: fig. 1F, ct; Jałoszyński 2025; Weide and Betz 2009: fig. 5). It is likely that this structure was reduced multiple times in the superfamily. The phylogenetic significance of the absence in Dasycerinae, Protopselaphinae and Pselaphinae is presently unclear due to fragmentary anatomical data.
13. Labral setae: (0) several long setae close to anterior margin; (1) setation largely reduced. A distinct vestiture of anterior labral setae is present in *Protopselaphus* (Newton and Thayer 1995) and most other groups of Staphylinidae (e.g., Blackwelder 1936; Thayer and Newton 1979: figs. 6 and 8), including most subgroups of Pselaphinae (e.g., Jeannel 1950). It is very sparse in *P. heisei*, and largely reduced or absent in Clavigeritae (Jałoszyński et al. 2020, 2022).
14. Antennal insertion: (0) exposed laterally or dorsally; (1) hidden below a shelf‐like frontal projection. The antennal insertion is exposed in staphylinoid outgroups (e.g., Blackwelder 1936; Antunes‐Carvalho et al. 2017) and also at least partly in most groups of Staphylinidae including *Protopselaphus* and *Dasycerus* (Newton and Thayer 1995: char. 17). It is always concealed below a shelf‐like frontal projection in Pselaphinae (Newton and Thayer 1995; Jałoszyński et al. 2020; 2022; Luo et al. 2021a; Beutel et al. 2021). This is a potential autapomorphy of the subfamily.
15. Basal articulatory piece of scapus: (0) visible; (1) not visible. Usually more or less exposed in Coleoptera (e.g., Blackwelder 1936: fig. 13) including Dasycerinae, *Protopselaphus* (Newton and Thayer 1995) and Faronitae (Jeannel 1950: fig. 2b). Absent in Pselaphitae and Clavigeritae according to Jeannel (1950: fig. 2a), but in fact deeply countersunk in the distal part of the scapus (Jałoszyński et al. 2020; 2022). This highly unusual condition, which is also documented in *P. heisei* and *Bergrothia* (Beutel et al. 2021; Luo et al. 2021a), is a potential synapomorphy of Pselaphinae excl. Faronitae (Jałoszyński et al. 2022).
16. Apical antennomere: (0) unmodified and apically rounded; (1) cone‐shaped and apically pointed; (2) club‐shaped and apically truncated. The terminal flagellomere is apically rounded and relatively short in *Protopselaphus* (Newton and Thayer 1995: fig. 6) and variable in extant and extinct Dasycerinae (Yin et al. 2021) and in *Glypholoma* (Thayer and Newton 1979: figs. 52–55). A more or less enlarged, cone‐shaped and apically pointed apical flagellomere occurs frequently in Pselaphinae (Beutel et al. 2021; Chandler 1991; Jałoszyński et al. 2022; Jeannel 1950: Figure 2a,b; Schomann et al. 2008: Fig. 10). This is a potential groundplan apomorphy of the subfamily even though this condition also occurs in other groups of Staphylinidae (Blackwelder 1936: Fig. 13). The terminal segment is large, probably composed of several flagellomeres, and apically truncated in Clavigeritae (Chandler 2001; Jałoszyński et al. 2020, 2022). As a whole, this character is so variable that the phylogenetic significance is minimal at best.
17. Size of antepenultimate antennomere: (0) not larger than preceding flagellomere; (1) distinctly larger than preceding flagellomere. Antennomere 9 is distinctly enlarged in Protopselaphinae (Newton and Thayer 1995). This is usually also the case in Pselaphinae, but it is only as large as antennomere 8 in *Faronus* (Jeannel 1950: fig. 2b). The condition varies strongly in Dasycerinae, in some species for instance with flagellomeres gradually increasing in size, but in some other cases with a moderately distinct 3‐segmented club (Yin et al. 2021). A specimen with a filiform antenna on one side and a club on the other is shown in Yin et al. (2021: Fig. 6).
18. Peduncles of flagellomeres: (0) present; (1) absent. Peduncles of antennomeres are very likely absent in the groundplan of Coleoptera and Staphyliniformia (e.g., Beutel et al. 2003, 2008; Dressler and Beutel 2010). The presence or absence in the groundplan of Staphylinoidea and Staphylinidae is ambiguous. They occur in many groups of rove beetles including Protopselaphinae, *Glypholoma* and Dasycerinae (Blackwelder 1936: fig. 12A–C; Thayer and Newton 1979: figs. 52–55; Newton and Thayer 1995: fig. 6; Yin et al. 2021), and also in Pselaphinae (e.g., Beutel et al. 2021; Jałoszyński et al. 2020, 2022; Jeannel 1950; Luo et al. 2021a). The absence of exposed membranes between antennomeres is arguably linked with this feature. Like the previous feature, this condition is highly variable and thus not useful in phylogenetic analyses.
19. Arrangement of antennal muscles: (0) with different areas of origin incl. anterior tentorial arms and not arranged parallel to the sagittal plane; (1) origin exclusively on vertical tentorial columns and arranged parallel to the sagittal plane. An unusual pattern of extrinsic antennal muscles is found in *Protopselaphus* and Pselaphinae, with all three originating on the vertical columns formed by the posterior and dorsal arms and with an arrangement parallel to the sagittal plane (Beutel et al. 2021; Jałoszyński et al. 2020, 2022; Luo et al. 2021a). This condition is unknown in other groups of Staphylinoidea with available anatomical data (Antunes‐Carvalho et al. 2017; Beutel et al. 2003; Weide and Betz 2009).
20. Distal region of mandible: (0) single tooth; (1) several teeth; (2) reduced. The distal region of the mandible is formed by a single well‐developed tooth in Protopselaphinae (Newton and Thayer 1995) and also in *Faronus lafertei* Aubé (Jeannel 1950: fig. 5b). A pointed apex and several subapical teeth are usually present in other groups of Pselaphinae (e.g., Beutel et al. 2021; Jeannel 1950: figs. 3 and 5c; Luo et al. 2021a; Schomann et al. 2008: figs. 18A–D, 19A), arguably a groundplan apomorphy of the subfamily excl. Faronitae. However, the distal mandibular region is distinctly reduced in the myrmecophilous genera *Diartiger* and *Claviger* (Jałoszyński et al. 2020, 2022: fig. 4a), arguably an autapomorphy of Clavigeritae.
21. Mesal mandibular edge: (0) with movable prostheca; (1) with elongate brush of microtrichia; (2) without prostheca or row of microtrichia. A prostheca developed as a movable semimembranous or sclerotized tooth is absent in most groups of Staphylinidae (Blackwelder 1936) including *Protopselaphus* (Newton and Thayer 1995: fig. 6) and Pselaphinae (Luo et al. 2021a: figs. 3A–D and 5A,B; Jeannel 1950: Figs. 3 and 5b,c). An immobile dense dorsomesal row of microtrichia is present on the mandible of *Protopselaphus*, as a vestigial prostheca (Lawrence et al. 2011). As numerous gradual modifications of the prostheca occur in Staphylinoidea (and other groups), this character is of very limited use in a phylogenetic context if at all.
22. Mandibular mola: (0) present; (1) distinctly reduced; (2) absent. It is present in the groundplan of Staphyliniformia and Staphylinoidea (Anton and Beutel 2004; Beutel et al. 2003; Beutel and Leschen 2005), and also well‐developed in some groups of Staphylinidae (Thayer and Newton 1979: fig. 8). Only a brush‐like structure is present in Dasycerinae (Newton and Thayer 1995). A mola is missing in *Protopselaphus* (Newton and Thayer 1995) and *P. heisei* (Beutel et al. 2021), but a vestige is present in *Bergrothia* and other pselaphines (Luo et al. 2021a: figs. 3A–D and 5a,b; Jeannel 1950; Jałoszyński et al. 2020, 2022). As a gradual reduction, this character is of minor phylogenetic significance.
23. Subdivision of galea: (0) present; (1) absent. Usually divided into a proximal and distal galeomere in Coleoptera including *Protopselaphus* and other staphylinids (Newton and Thayer 1995: Fig. 4; Thayer and Newton 1979: Fig. 4; Beutel et al. 2003; Anton and Beutel 2004; Lawrence et al. 2011). It is also divided in *P. heisei* and other pselaphines even though this condition can be difficult to observe (e.g., Jeannel 1950: Figs. 4a and 5a,c).
24. Size of penultimate maxillary palpomere: (0) not enlarged, similar in size to preceding palpomere; (1) distinctly enlarged and club‐shaped; (2) short articulatory piece. Distinctly enlarged and club‐shaped in *Protopselaphus* (Newton and Thayer 1995: figs. 2 and 4). Short and triangular in *P. heisei* (Beutel et al. 2021) and also very short in most other groups of Pselaphinae with well‐developed maxillary palps (Jeannel 1950; Chandler 2001; Luo et al. 2021a), but conspicuously enlarged in many Ctenistini, large in Thaumastocephalini (Jałoszyński et al. 2023), and nearly as long as palpomere 2 in some Metopiasini. A potential autapomorphy of the subfamily, with possible cases of secondary modifications.
25. Apical sensillum (sensory appendage) of ultimate maxillary palpomere: (0) absent; (1) present. Absent in *Protopselaphus* (Newton and Thayer 1995: fig. 4) and other groups of Staphylinidae (Blackwelder 1936; Spiessberger et al. 2024). Present in all Pselaphinae, although in some cases small and inconspicuous (e.g., Chandler 2001; Jeannel 1950; Luo et al. 2021a). Probably a groundplan apomorphy of the subfamily.
26. Exposure of the posterior submentum; (0) fully exposed externally; (1) exposed as narrow longitudinal stripe or ‘submental carina’ (“gular carina”; Chandler 1991); (2) completely absent from the surface due to medially confluent genae. The posterior submentum in Pselaphinae is sometimes totally absent from the ventral cephalic surface due to genal extensions meeting in the midline, as for instance in species of *Faronus*. A narrow stripe of the posterior submentum is exposed in *Brachygluta* of Goniaceritae and in other groups. The distinct median extension of the genae appears to be a typical feature of the Omaliine Group, but with a considerable degree of variation even within Pselaphinae. For instance, in at least some Jubini the entire submentum is laterally delimited by complete hypostomal sulci.
27. Shape of mentum: (0) transverse; (1) subquadrate to elongate. The mentum is usually transverse in Staphylinidae (e.g., Blackwelder 1936; Thayer and Newton 1979) but subquadrate to elongate in *Protopselaphus* and Pselaphinae according to Newton and Thayer (1995: char. 48) (see also Beutel et al. 2021; Luo et al. 2021a). However, the mentum of *Faronus* is wider than long (Jeannel 1950: fig. 3c). This character requires a re‐evaluation.
28. Exposure of prementum: (0) distinct and externally visible; (1) largely concealed below mentum. The prementum is largely exposed in *Protopselaphus* (Newton and Thayer 1995) and other groups of Staphylinidae including various groups of Pselaphinae (e.g., Jeannel 1950). It is largely or completely concealed below the mentum in *P. heisei* (Beutel et al. 2021), *Bergrothia* (Luo et al. 2021a: figs. 1C and 4B), and also in Clavigeritae (Jałoszyński et al. 2020, 2022). The condition can be affected by shrinking artefacts concerning the membrane connecting the mentum and prementum.
29. Lateral hypopharyngeal lobes (superlinguae): (0) directed anterad and meeting at middle; (1) directed anterolaterad and separated. These lateral hypopharyngeal lobes, addressed as “paraglossae” by some authors (see e.g., Spiessberger et al. 2024), are present as prominent flat structures with a glabrous ventral surface in *Protopselaphus* (Newton and Thayer 1995: fig. 5), directed anterad and touching at middle, possibly an autapomorphy of Protopselaphinae. In Pselaphinae, they are short in *Faronus* (Jeannel 1950: fig. 5a) but almost as long as the mentum in *Bergrothia* (Luo et al. 2021a: fig. 5F), and also elongated in *Batrisus* and *Euplectus* (Jeannel 1950: figs. 4 and 5), and diverging anterolaterad, so that the mesal margins of the lobes are widely separated. This feature varies very strongly in Staphylinidae (Spiessberger et al. 2024). Paraglossae are generally absent in Holometabola excl. Hymenoptera, i.e., Aparaglossata (Beutel et al. 2013; Jałoszyński 2025).
30. Terminal (third) labial palpomere: (0) large; (1) conspicuously reduced. In Pselaphinae the labial palpomere 3 is extremely small, papillate (in some Euplectitae), minute and rod‐like (in many Faronitae and higher Pselaphinae), or setiform, similar to a thick bristle and can be mistaken for one of the apical setae of palpomere 2 (many higher Pselaphinae, except for Clavigeritae, which have lost the palps). In *Protopselaphus* it is clearly thicker than in any Pselaphinae and moderately large, similar to the condition found in many other Staphylinidae. The shape of the terminal labial palpomere within Staphylinidae varies distinctly, but it is always clearly larger than in Pselaphinae.
31. Composition of M. frontopharyngalis posterior (M. 46): (0) two separate subcomponents, one (M. 46b) with a diagonal orientation; (1) one nearly vertical subcomponent (M. 46a). M. 46b with a diagonal orientation is present in most of the species examined by Weide and Betz (2009) and in *Protopselaphus* but missing in all species of Pselaphinae examined so far (Beutel et al. 2021; Jałoszyński et al. 2020, 2022; Luo et al. 2021a).
32. Position of the brain: (0) anterior to neck region; (1) in neck region. The supra‐ and suboesophageal ganglionic complexes are located in the narrowed neck region in *Protopselaphus* and all examined representatives of Pselaphinae, and almost completely fill out this space (Beutel et al. 2021; Jałoszyński et al. 2020, 2022; Luo et al. 2021a). This derived condition is also present in Dasycerinae, but no data are available for other members of the Omaliine Group.
33. Ventral pharyngeal dilators: (0) well‐developed; (1) only single muscle (M. 50) extending from posteroventral to anteroventral pharynx. Whereas the posterior pharyngeal dilators are strongly developed in Pselaphinae (Beutel et al. 2021; Jałoszyński et al. 2020, 2022; Luo et al. 2021a), only a single intrinsic ventral bundle is present in *Protopselaphus*.
34. Position of the frontal ganglion: (0) directly above anatomical mouth; (1) distinctly posterior to anatomical mouth. The frontal ganglion is a very constant landmark indicating the border between the prepharynx and pharynx in Coleoptera and insects in general (e.g., Beutel et al. 2008; 2021; Dressler and Beutel 2010; Jałoszyński et al. 2020; 2022; Luo et al. 2021a). The posterior shift of the frontal ganglion in *Protopselaphus* is thus a highly unusual feature and likely an autapomorphy.
35. Labral glands: (0) absent; (1) present. Well‐developed paired glands are present in the anterior head region of *P. heisei*, *Bergrothia*, *Claviger* and *Diartiger* (Beutel et al. 2021; Luo et al. 2021a; Jałoszyński et al. 2020). They are absent in *Protopselaphus* and information on Faronitae is not available at present. The glands are very likely absent in the groundplan of Staphylinoidea (Antunes‐Carvalho et al. 2017; Beutel et al. 2003; Jałoszyński 2025; M. I. Yavorskaya et al. 2023) and Staphylinidae (Evans 1964).
36. Labio‐hypopharyngeal glands: (0) absent; (1) present. More or less strongly developed labio‐hypopharyngeal glands are present in the anterior head region of *Protopselaphus* and all pselaphine species with available anatomical data (Beutel et al. 2021; Jałoszyński et al. 2020, 2022; Luo et al. 2021a). This is a potential synapomorphy of both subfamilies. However, in contrast to abdominal defensive glands (e.g., Dettner 1993), data on cephalic glands of Staphylinidae are almost completely lacking (e.g., Evans 1964), including Faronitae. The labio‐hypopharyngeal glands are likely absent in the groundplan of Staphylinoidea (Antunes‐Carvalho et al. 2017; Beutel et al. 2003; Jałoszyński et al. 2025; Yavorskaya et al. 2023). Very large glands are present in the posterior head region and also in the anterior thorax of *Dasycerus sulcatus*.
37. Cervical sclerites: (0) distinctly developed; (1) vestigial or absent. The lateral cervical sclerites or laterocervicalia are distinctly developed in most groups of Staphylinidae (Naomi 1987; Newton and Thayer 1995: char. 25), but largely or completely reduced in Protopselaphinae and Pselaphinae, and also in Dasycerinae and Micropeplinae (Newton and Thayer 1995). Our unpublished observations show that a pair of cervical sclerites occurs in Pselaphinae, but they are small, situated close to the posterior head margin, weakly sclerotized and difficult to observe even with SEM.

## 5 Discussion

Our study on the cephalic anatomy of *Protopselaphus* sp. from Thailand supports the phylogenetic interpretation of Newton and Thayer (1995), who suggested *Protopselaphus* as a link between the highly derived and specialized Pselaphinae and related lineages of rove beetles of the Omaliine Group. The head of *Protopselaphus* shows a remarkable combination of plesiomorphic character states absent in Pselaphinae, and some derived features shared with this specialized subfamily.

### 5.1 Phylogeny

The sister group relationship between Protopselaphinae and Pselaphinae was established by Newton and Thayer (1995) and this is confirmed by our results. The clade is supported by an entire series of cephalic synapomorphies, including the following features: compound eye in lateral view situated in the ventral half of the head (homoplasious, eyes mostly located at middle region in Faronitae), massive vertical tentorial columns, complete separation of the tentorial bridge from the rest of the tentorium (Figure 6), distinct dorsal tentorial pits internally connected with the dorsal arms, extrinsic antennal muscles with an arrangement parallel to the sagittal plane, and large and rather compact labio‐hypopharyngeal glands. An additional shared derived feature is a shift of the brain to the narrowed neck region and filling out this space almost completely. However, this condition is also present in *Dasycerus sulcatus*, and occurs also outside the Omaliine Group (in some Scydmaeninae; Jałoszyński et al. 2012). The fusion of the posterior tentorial pits, shared by *Protopselaphus* and many Pselaphinae, is a feature of unclear phylogenetic value. This condition occurs in most Euplectitae, Batrisitae and some Pselaphitae, but the pits are generally separated in Faronitae and Goniaceritae (unpublished obs. P. Jałoszyński). However, the posterior tentorial pits in Pselaphinae, when separated, are mostly circular, and it seems that in those pselaphines which have the pits connected, and also in *Protopselaphus*, the fusion has occurred between circular pits. In all remaining members of the Omaliine Group the posterior tentorial pits are strikingly different, elongate, usually very narrow and slot‐like (if discernible at all). Only a moderate elongation occurs in *Dasycerus*, so that each pit is comma‐shaped. It is conceivable that the circular pits are a synapomorphy of Protopselaphinae + Pselaphinae, and those in *Dasycerus* represent a transitional state between these two subfamilies and the rest of the Omaliine Group. Moreover, the median pit on the frontovertexal region in *Protopselaphus*, even though shallow, resembles a similarly placed pit present in some Faronitae, for example, *Faronus*. The suggested synapomorphies of Protopselaphinae and Pselaphinae refute arguments of Kurbatov (2024), who rejected a close relationship between both subfamilies. However, it is evident that other body parts must be examined and a comprehensive set of characters included in phylogenetic analyses with a wide selection of outgroups in order to better understand relationships of subfamilies of rove beetles. Moreover, anatomical data on members of the Omaliine Group and other staphylinid lineages are still scarce.

The monophyly of the monogeneric Protopselaphinae has never been questioned. Cephalic autapomorphic conditions are anterolateral triangular projections of the mentum separated by a deep median emargination, maxillary palpomere 4 with two elongate and flattened ventral subapical sensilla, the anteriorly projecting and mesally adjacent superlinguae of the hypopharynx, the posterior shift of the frontal ganglion, and the presence of only a single ventral pharyngeal dilator, with origin and insertion on the pharynx. The ring of pores at the bottom of each dorsal tentorial pit is another potential autapomorphy of Protopselaphinae, but the presence of this character was not examined in other species except for *Protopselaphus watrousi* Newton & Thayer (examined by PJ). This structure deserves more attention, as it may suggest a possible glandular function for dorsal cephalic foveae, one of the landmark features of Pselaphinae.

Based on our previous investigations, Pselaphinae are strongly supported by cephalic apomorphies, such as a thin filament‐like (or absent) anterior tentorial arm, an antennal insertion completely covered by a frontal projection, a distinctly developed paired labral gland, a strongly reduced labial palpomere 3, a thin apical sensillum on the maxillary palpomere 4, and a posterior frontopharyngeal muscle (M46) composed of only one subcomponent (M 46b absent). The latter, as well as other changes in the muscular system, can be linked to drastic changes of the shape of the head and inner skeletal elements. The foveal system, a conspicuous derived character complex of Pselaphinae (e.g., Chandler 2001; Jeannel 1950), is another potential autapomorphy. However, the pattern varies very strongly within the group, and foveae also occur in other staphylinid lineages, for instance in Scydmaeninae (Jałoszyński 2025) or in Dasycerinae (Yin et al. 2021).

A clade comprising Dasycerinae and Protopselaphinae + Pselaphinae, as already suggested by Newton and Thayer (1995), is only weakly supported by our results, partly due to lacking anatomical data of the former group. A shared derived condition is the absence of the laminatentorium (Figure 6), but it is possibly also missing in other members of the Omaliine Group. Clearly, more detailed investigations on the morphology of Dasycerinae and other potentially related taxa is required for a robust morphology‐based phylogenetic placement of the subfamily. Some ventral foveae on thoracic areas are shared by *Dasycerus* and Pselaphinae. However, they are missing in *Protopselaphus* (Newton and Thayer 1995).

### 5.2 Transition to Predaceous Feeding Habits

Cephalic character transformations have apparently played a major role in the evolution of the lineage that comprises Protopselaphinae and Pselaphinae. This includes changes in the shape of the head capsule, a distinctly modified configuration of the tentorium, modifications of the mouthparts, and acquisition of cephalic glands. One of the reasons for those transformations is most likely a switch to predacious habits, that clearly distinguish the two subfamilies from the mycophagous Dasycerinae (Yin et al. 2021). In this context it should be mentioned that glands of the head and other body regions may have evolved earlier, in this case not associated with predacious habits. It is also noteworthy that very strongly developed cephalic and thoracic glands are present in *Dasycerus sulcatus*, with glandular openings at the tips of the multiple setiferous tubercles, and that a large part of the body surface is covered with secretions in this species, very likely with the function to form a camouflage mechanism together with small substrate particles.

The neck region in most of the pselaphines is semi‐globular and separated from the remaining head capsule by a very distinct occipital constriction, and forms a ball‐and‐socket joint with the anterior prothorax. Although the occipital constriction is less distinct in *Protopselaphus*, the articulation between the posterior cephalic region and the prothorax is similar to the condition observed in Pselaphinae. A similar cephalic structure in phylogenetically remotely related Scydmaeninae was interpreted as increasing the mobility of the head related to predation on small arthropods (Jałoszyński 2025).

As already shown in Newton and Thayer (1995), the feeding apparatus of *Protopselaphus* shows a distinctive combination of characteristics typical for predators on one hand, and features found in fungivorous and detritophagous beetles on the other. Although the mandibles in *Protopselaphus* are not of a typical falcate shape as in most pselaphines and lack the subapical teeth, their slender and apically curved shape and the lack of a mola with a grinding surface suggest that they may be used for grasping small prey. A grinding mola is distinctly developed in *Dasycerus* and other mycophagous staphylinids. Another feature that *Protopselaphus* shares with Pselaphinae is an unusually thin mandibular abductor (pers. obs. M. Yavorskaya, various pselaphine species). This also suggests a switch from chewing to grasping, since this muscle is well developed in beetles with saprophagous or mycophagous feeding preferences (Yavorskaya et al. 2017), for instance in *Dasycerus sulcatus*. The presence of a brush‐like prostheca and partially preserved smooth molar lobes, as well as rows of microtrichia on the epipharynx and hypopharynx and fringes of setae on the anterior rim of the labrum and prementum tentatively suggest that microphagy or liquid feeding might also be involved, as already suggested by Newton and Thayer (1995). However, a reliable assessment of the feeding habits in Pselaphinae is still a challenging task. Enlarged, often hypertrophied maxillary palps that can be involved in prey grasping (Schomann et al. 2008) are a signature feature of Pselaphinae (*psēlapháō* – to feel, handle, touch). *Protopselaphus* also has rather large maxillary palps, presumably with well‐developed intrinsic and extrinsic musculature, but their involvement in prey seizure seems unlikely. Maxillary palps showing a similar relative size and structure can be found in Scydmaeninae (Stenichnini), which are known to use adhesive protibial setae, the labium, and mandibles to grasp prey, while the palps are only involved as sensory organs (e.g., Jałoszyński and Olszanowski 2013). Although specialized predation has been an ongoing trend in most of the major pselaphine lineages (Beutel et al. 2021), a rather small but distinct molar region with dense rows of sclerotized cuticular teeth occurs in multiple lineages of the subfamily. This also includes Faronitae and is thus probably part of the groundplan. In contrast, the mola is completely reduced in other predaceous staphylinid groups (e.g., Spiessberger et al. 2024). Live observations and studies on the functional morphology of the feeding apparatus of *Protopselaphus* and Pselaphinae are needed for a better understanding of the function of the structures and features discussed here.

### 5.3 Mechanical Stabilization

The mechanical stabilization of the head with a relatively thick cuticle (Figure 6C) and a fairly narrow lumen, and massive dorsoventral tentorial columns, is a syndrome that may have improved protection against ground‐oriented predators such as ground beetles or certain ant species. This may also apply to the labio‐hypopharyngeal glands. Even though the composition and function of the secretions are still unexplored, it is conceivable that they may be defensive. A fascinating aspect of Pselaphinae is that myrmecophilous habits evolved multiple times independently in this subfamily (Chandler 2001; Jałoszyński et al. 2022; Park 1947; Parker and Grimaldi 2014), and the two syndromes outlined above may have served as preadaptations for such a highly specialized lifestyle. Anatomical and behavioral studies on different pselaphines are necessary for understanding why such strong mechanical reinforcements and glandular systems became necessary in the early evolutionary history of Pselaphinae.

## 6 Conclusions

The anatomical data we present confirm that *Protopselaphus* is the sister taxon of Pselaphinae. This concept is supported by an entire series of cephalic synapomorphies, and a close relationship of Dasycerinae is at least tentatively suggested. We propose that predacious habits combined with facultative microphagy or liquid feeding is part of the groundplan of the well‐established clade Protopselaphinae + Pselaphinae, with a more pronounced preference for carnivory in the latter group. A typical syndrome is mechanical reinforcement of the head and other body regions, e.g., column‐like tentorial elements and foveae, combined with complex cephalic glands. This may have resulted in better protection against predators in leaf litter habitats, and also formed preadaptations for myrmecophilous habits, which evolved in different lineages of Pselaphinae.

## Author Contributions

**Margarita I. Yavorskaya:** conceptualization, methodology, software, data curation, investigation, formal analysis, funding acquisition, writing – original draft, writing – review and editing, visualization, project administration. **Paweł Jałoszyński:** writing – original draft, writing – review and editing, resources, formal analysis, investigation. **Shûhei Nomura:** resources, writing – review and editing. **Rolf G. Beutel:** conceptualization, writing – review and editing, writing – original draft, formal analysis, investigation.

## Data Availability

The data that support the findings of this study are openly available in Zenodo at https://zenodo.org/
